# Long-term explantation risk in patients with chronic pain treated with spinal cord or dorsal root ganglion stimulation

**DOI:** 10.1136/rapm-2024-105719

**Published:** 2024-07-30

**Authors:** Kliment Gatzinsky, Beatrice Brink, Kristin Lilja Eyglóardóttir, Tobias Hallén

**Affiliations:** 1 Department of Neurosurgery, Sahlgrenska University Hospital, Gothenburg, Sweden; 2 Department of Clinical Neuroscience, University of Gothenburg Institute of Neuroscience and Physiology, Goteborg, Sweden

**Keywords:** Spinal Cord Stimulation, CHRONIC PAIN, Treatment Outcome

## Abstract

**Objective:**

To investigate long-term explantation risks and causes for the explantation of neuromodulation devices for the treatment of chronic pain from different manufacturers.

**Methods:**

This retrospective analysis included patients implanted with a system for spinal cord stimulation (SCS) or dorsal root ganglion (DRG) stimulation at Sahlgrenska University Hospital between January 2012 and December 2022. Patient characteristics, explantation rates and causes for explantation were obtained by reviewing medical records.

**Results:**

In total, 400 patients were included in the study. Including all manufacturers, the cumulative explantation risk for any reason was 17%, 23% and 38% at 3, 5 and 10 years, respectively. Explantation risk due to diminished pain relief at the same intervals was 10%, 14% and 23%. A subgroup comparison of 5-year explantation risk using Kaplan-Meier analysis did not show a statistically significant difference between the manufacturers. In multivariable Cox regression analyses, there was no difference in explantation risk for any reason, but for explantation due to diminished pain relief, a higher risk was noted for Medtronic (preferably older types of SCS devices) and DRG stimulation. No other predictive factor for explantation was found.

**Conclusions:**

Although SCS and DRG stimulation are well-established and safe treatments for chronic pain, the long-term explantation risk remains high. The difference between manufacturers highlights the importance of technological evolution for improving therapy outcomes. Increased stringency in patient selection and follow-up strategies, as well as further development of device hardware and software technology for increased longevity, could possibly reduce long-term explantation risks.

**What is already known on this topic**

- Reported risks of neuromodulation system explantation in the treatment of chronic pain vary greatly in the literature, and real-world data regarding long-term explantation risk (≥5 years) are limited.

**What this study adds**

- This study adds objective, real-world data showing that long-term explantation risk is not negligible. The possible differences between manufacturers emphasize the importance of continuous technical development for improving clinical outcomes.

**How this study might affect research, practice or policy**

- Further comparative, non-manufacturer-funded, prospective long-term studies are needed to scrutinize the differences in effect between different treatment modalities from spinal cord stimulation manufacturers. In order to minimize explantation rates, increased stringency regarding patient selection and the improvement of neuromodulation device hardware and software for increased therapy longevity have to be implemented.

## Introduction

 Spinal cord stimulation (SCS) is a treatment option used to alleviate chronic pain in patients with various conditions, such as postoperative low back and leg pain, complex regional pain syndrome, neuropathic pain syndrome and ischemic pain syndrome.^1^ SCS was first introduced in the 1960s, and since then, the treatment has become an important tool in managing pain unresponsive to surgery or to medication and other conservative therapies.^2 3^ SCS is generally considered safe and effective when used correctly in carefully selected patients.^4^ Studies have demonstrated not only a reduction in pain but also improvements regarding quality of life (QoL) and functional status.^2^ There are several randomized controlled trials (RCTs) indicating the treatment efficacy of SCS.^5^,^10^ However, the robustness of the level of evidence for SCS has been questioned in recent reviews and studies.^11^,^13^

Due to the difficulties of generalizing the results of RCTs to the clinical setting, the use of real-world data has become an important tool to study the long-term effects of SCS.^14 15^ Although there are few severe complications associated with SCS, the overall complication rate remains 20%–40%, including infection and technical issues related to the lead and implantable pulse generator (IPG).^16 17^ In addition to complications, diminished pain relief could result in the explantation of a SCS system. Previous studies report explantation rates ranging from 4% to 40%, depending on the follow-up time, the reported reason for explantation and direct manufacturer involvement in the study, that is, data from manufacturer-sponsored registries.^1518^,^28^ There are several different manufacturers providing SCS systems, and manufacturer-independent studies are needed to compare long-term outcomes, including explantation rates. The objective of this study is (1) to investigate the overall 10-year risk of explantation and (2) to assess the 5-year explantation risk and causes of explantation of devices used for SCS and dorsal root ganglion (DRG) stimulation from various manufacturers.

## Materials and methods

### Patients

This single-center study includes all patients who, after 2–3 weeks of test trials, received a permanent implant of an SCS or DRG stimulation system from the manufacturers Abbott (8701 Bee Caves Rd, Austin, Texas, USA), Boston Scientific (300 Boston Scientific Way, Marlborough, Massachusetts, USA), Medtronic (710 Medtronic Parkway, Minneapolis, Minnesota, USA), Nevro (1800 Bridge Parkway, Redwood City, California, USA) or Saluda (9401 James Ave, Bloomington, Minnesota, USA) at Sahlgrenska University Hospital between January 1, 2012 and December 31, 2022. Implanted patients were identified by computerized operating lists. The percutaneous implantation technique with permanent leads was used in all patients with implants performed by four neurosurgeons with extensive experience in SCS for the treatment of chronic pain and by one neurosurgeon for DRG stimulation devices. Medical records were reviewed until December 31, 2023, allowing for patients to be followed up for a minimum of 1 year. Information about the manufacturer that provided the SCS system, date of implantation, date of explantation, if applicable, and cause leading to explantation was collected. Patient characteristics were registered considering gender, age at implantation, indication for the implant, presence of diabetes mellitus, immunosuppressive treatment (peroral corticosteroids, disease-modifying antirheumatic drugs or biologic disease-modifying antirheumatic drugs) and whether a rechargeable or a non-rechargeable IPG was implanted. Computerized operating lists and electronic medical records were searched by two of the authors and cross-checked to ensure that no adequate data were left out.

Causes leading to explantation were classified as ineffective pain relief, conditions requiring MRI, infection, frequent dislocation, no longer any pain requiring SCS treatment, uncomfortable stimulation or IPG pocket pain. Surgery to replace a depleted IPG was not classified as an explantation. Two different analyses were then performed. First, an overall assessment of the 10-year explantation risk, including all manufacturers, was made. Second, a subgroup analysis for each of the investigated manufacturers regarding the 5-year explantation risk was carried out. The rationale for this was that at Sahlgrenska University Hospital, the use of SCS devices from the different manufacturers has varied over time, with more use of Abbott and Medtronic products at the beginning of the study period and Boston Scientific and Nevro at the later stages of the study period, which leads to discrepancies considering the possibility for long-term follow-up between the manufacturers. Accordingly, the analysis of explantation risks was based on a follow-up time of up to 5 years in order to maximize the number of included patients from the different manufacturers. Only five patients were implanted with Saluda devices and none of these had a follow-up exceeding 2 years. This manufacturer was therefore excluded from the comparative subgroup analysis regarding 5-year explantation risk.

### Ethics

The study was approved by the Swedish Ethical Review Authority (Dnr 2023-01730-01).

### Statistics

Patient characteristics are presented with categorical data as numbers and percentages; normally distributed data are presented as mean±SD, and non-normally distributed data are presented as median and IQR. Survival analysis, including Kaplan-Meier curves, is used to visualize explantation risks, including log-rank tests to compare explantation risks between the manufacturers. Differences in 5-year explantation risks between the manufacturers were also analyzed with univariable and multivariable Cox regression analyses in order to adjust for other variables with possible influences on explantation. All tests were two-sided and significant results were defined at the level of p<0.05. Analyses were performed using SPSS (V.29.0; IBM Corp, Armonk, New York, USA).

## Results

A total of 400 patients were implanted during the study period, of whom 37% received a device from Boston Scientific, 27.5% from Medtronic, 22.5% from Nevro, 11.8% from Abbott and 1.3% from Saluda. 16 of 47 patients (34%) implanted with a device from Abbott received a system for DRG stimulation. The mean age at implantation was 51.8±12.5 years, and the majority of the patients were female (58.8%). In addition, 6.8% of the patients had diabetes mellitus and 2.8% had immunosuppressive treatment. The most common indication for implantation was persistent spinal pain syndrome type 2 (PSPS2), formerly failed back surgery syndrome, in 61% of the treated patients (table 1).

**Table 1:** Patient characteristics for the whole cohort

Implants	400
Explantations	96
Explant reasons, No. (% of explantations)	
Diminished pain relief	53 (55.2)
Conditions requiring MRI	17 (17.7)
Infection	8 (8.3)
Frequent dislocation	1 (1.0)
IPG pocket pain	2 (2.1)
No longer pain	4 (4.2)
Other reason	11 (11.5)
Age, years (mean±SD)	51.8±12.5
Gender, No. (%)	
Male	165 (41.2)
Female	235 (58.8)
Indication for implantation, No. (%)	
PSPS2	244 (61.0)
PSPS1	19 (4.8)
Peripheral neuralgia	47 (11.8)
Intercostal neuralgia	17 (4.3)
Polyneuropathy	12 (3.0)
CRPS	41 (10.3)
Other	20 (5.0)
Follow-up time, months (median, IQR)	47.5 (25-74)
Time to explantation, months (median, IQR)	26 (14-45)
SCS system, No. (%)	
Boston Scientific	148 (37.0)
Medtronic	110 (27.5)
Nevro	90 (22.5)
Abbott	47 (11.8)
Saluda	5 (1.3)
Rechargeable, No. (%)	
Yes	272 (68.0)
No	128 (32.0)
DRG stimulation, No. (%)	16 (4.0)
Diabetes mellitus, No. (%)	
Yes	27 (6.8)
No	373 (93.2)
Immunosuppression treatment, No. (%)	
Yes	11 (2.8)
No	389 (97.2)

CRPS, complex regional pain syndrome; DRG, dorsal root ganglion; IPG, implantable pulse generator; PSPS1, persistent spinal pain syndrome type 1; PSPS2, persistent spinal pain syndrome type 2; SCS, spinal cord stimulation.

**Figure 1:**
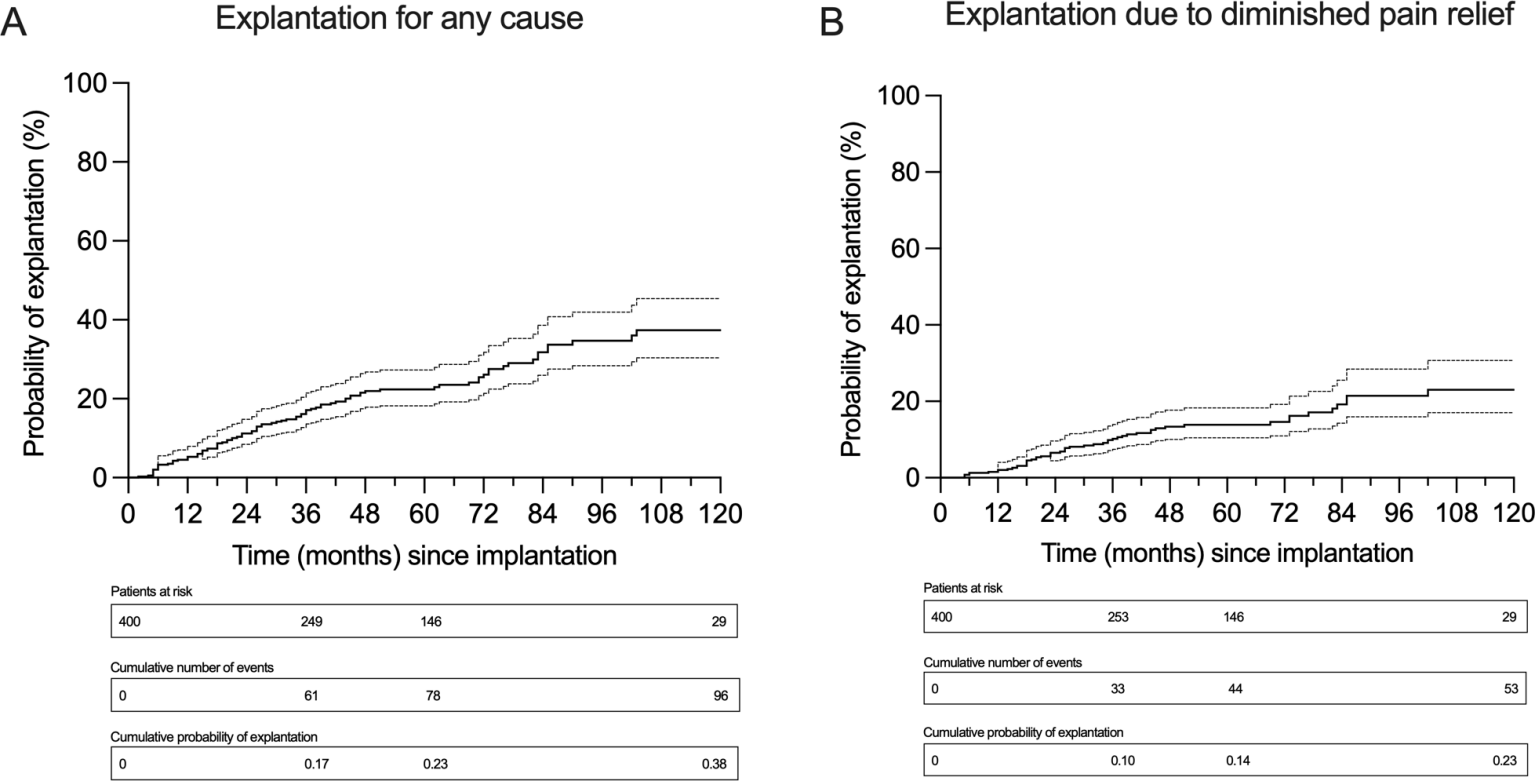
Survival analysis of all 400 patients with a Kaplan-Meier curve with a 95% CI, including patients at risk, the cumulative numbers of events and the probability of explantation at 3, 5 and 10 years, all manufacturers included. (A) Explantation for any cause and (B) explantation due to diminished pain relief.

In total, 96 patients (24%) had their stimulation device explanted during the study period. The median time to explantation was 26 months (IQR 14–45). Of the explanted devices, 55.2% of explantations were due to diminished pain relief, 17.7% were due to the need for MRI and 8.3% were due to infection (corresponding to 13%, 4%, and 2% of all 400 implantations, respectively) (table 1). Of the 96 explantations, 72 were therapy-ending. For 24 patients, reimplantation was performed after explantation: 7 of 17 (41%) after MRI requirement, 5 of 8 (63%) after infection, 2 of 11 (18%) after experiencing uncomfortable stimulation and 10 of 53 (19%) after diminished pain relief. In 15 of 24 (63%) reimplantations, a system from a different manufacturer was chosen based on the possibility of testing other stimulation modalities. The overall cumulated risk of explantation for any reason, including all manufacturers, was calculated to be 17%, 23% and 38% at 3, 5 and 10 years, respectively (figure 1A). Explantation risk due to diminished pain relief at the same intervals was 10%, 14% and 23% (figure 1B).

### 5-year explantation risk for the different manufacturers

**Figure 2:**
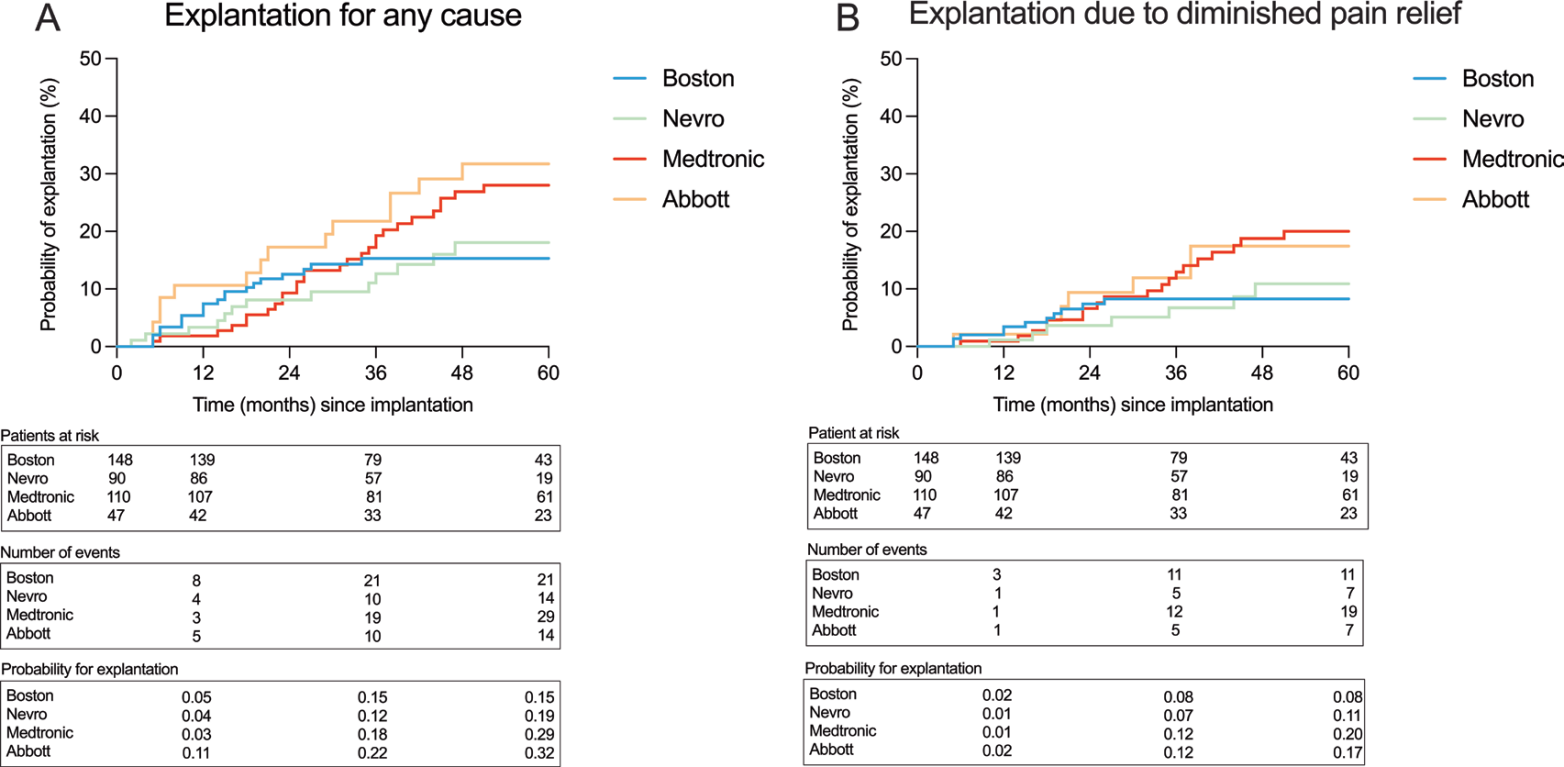
Survival analysis of 395 patients (five Saluda patients excluded) with Kaplan-Meier curves including patients at risk, the cumulative numbers of events and the probability of explantation at 1, 3 and 5 years for the four major included manufacturers. A comparison of curves for 5-year explantation risk was made with the log-rank (Mantel-Cox) test. (A) Explantation for any cause (p=0.14) and (B) explantation due to diminished pain relief (p=0.19).

The 5-year explantation risk for any reason for the different manufacturers was 15%, 19%, 29% and 32% for Boston Scientific, Nevro, Medtronic and Abbott, respectively (log-rank p=0.14, figure 2A). The 5-year explantation risk due to diminished pain relief was 8%, 11%, 20% and 17%, respectively (log-rank p=0.19) (figure 2B).

In Cox regression analyses, there was no statistically significant difference in 5-year explantation risk for any causes between the manufacturers in the univariable or multivariable analyses (data not shown). Regression analysis for explantation due to diminished pain relief did not show any statistically significant difference between the manufacturers in the univariable analysis, but higher risk for Medtronic devices (HR 2.72; 95% CI 1.18 to 6.3; p=0.02) in the multivariable analysis and for DRG stimulation in both univariable and multivariable analyses (HR 3.03; 95% CI 1.20 to 7.71; p=0.02 and HR 6.73; 95% CI 1.26 to 35.88; p=0.03, respectively) (table 2). No other variable was a significant predictor for explantation in the univariable or multivariable analyses, including the type of indication for implantation (table 2).

**Table 2:** Univariable and multivariable Cox regression analyses of potential predictors for 5-year explantation risks due to diminished pain relief; 395 patients (five Saluda patients excluded)

Variable	Univariable analysis			Multivariable analysis		
	HR	95% CI	P value	HR	95% CI	P value
Age (years)	1.00	0.98 to 1.03	0.80	1.00	0.97 to 1.03	0.99
Gender						
Male	1*	–		1	–	
Female	1.07	0.59 to 1.96	0.82	1.21	0.64 to 2.26	0.56
Indication						
PSPS2	1			1	–	
Other	0.81	0.44 to 1.52	0.52	0.64	0.31 to 1.35	0.24
Diabetes						
No	1	–		1	–	
Yes	1.50	0.53 to 4.17	0.45	1.74	0.58 to 5.23	0.32
Immunosuppressive treatment						
No	1	–		1	–	
Yes	2.41	0.58 to 9.96	0.23	2.46	0.57 to 10.65	0.23
Company						
Boston Scientific	1	–		1	–	
Nevro	0.98	0.38 to 2.53	0.97	0.81	0.30 to 2.17	0.68
Medtronic	1.96	0.93 to 4.11	0.08	2.72	1.18 to 6.30	**0.02**
Abbott	1.79	0.70 to 4.6	0.23	1.56	0.31 to 7.83	0.59
Rechargeable						
No	1	–		1	–	
Yes	0.91	0.49 to 1.67	0.76	2.21	0.97 to 5.07	0.06
DRG stimulation						
No	1	–		1		
Yes	3.03	1.20 to 7.71	**0.02**	6.73	1.26 to 35.88	**0.03**

* Reference category. Significant numbers are in bold.

DRG, dorsal root ganglion; PSPS2, persistent spinal pain syndrome type 2.

A separate analysis of the risk of explantation of DRG stimulation devices showed that 8 of 16 devices, which were all implanted to treat conditions with focal neuropathic pain, were explanted during the whole study period, rendering a 3-year, 5-year and 10-year accumulated explantation risk of 31%, 38% and 63%, respectively. The median time to explantation was 21 months (IQR 12–57). Five explantations were due to diminished pain relief, two because of lead dysfunction and one because of the need for an MRI.

**Figure 3:**
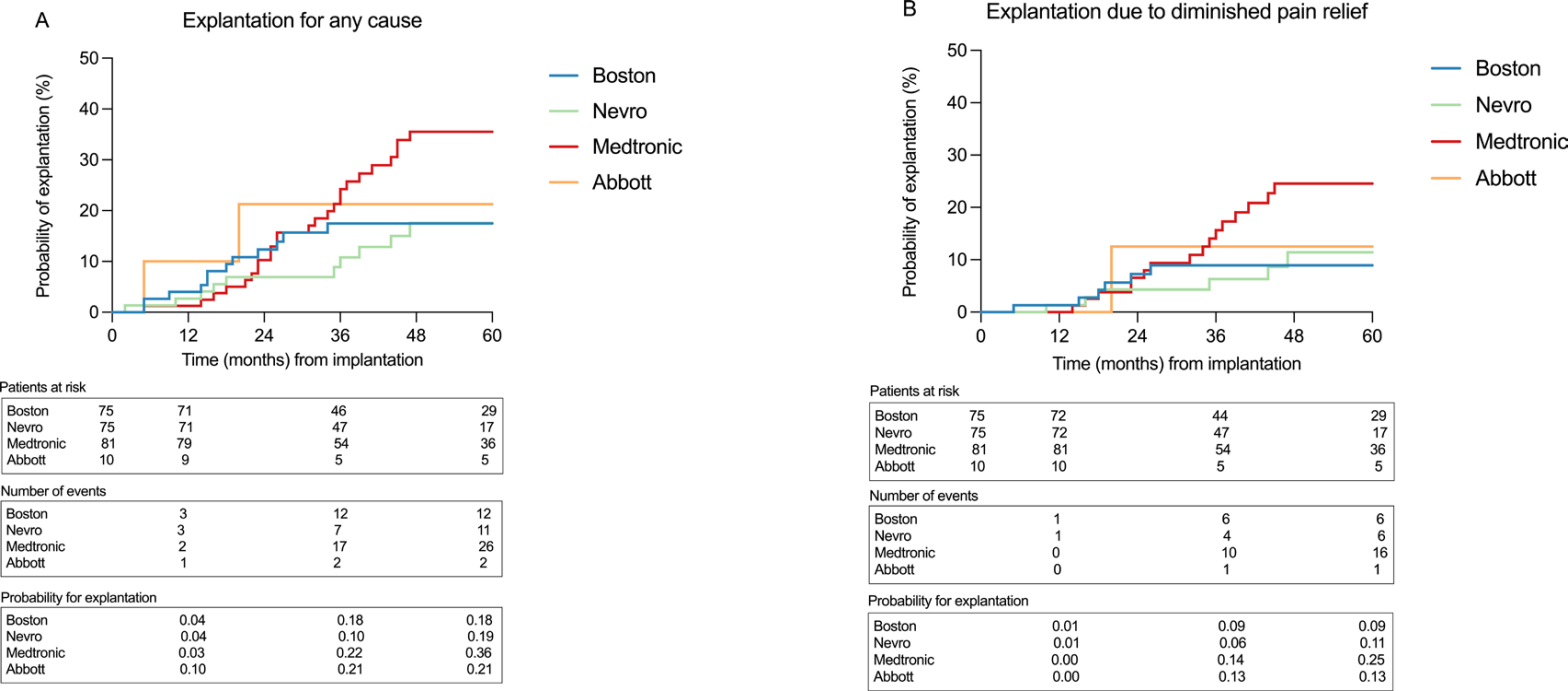
Subgroup survival analysis for the 241 patients with PSPS2 (three Saluda patients excluded) with Kaplan-Meier curves including patients at risk, the cumulative numbers of events and the probability of explantation at 1, 3 and 5 years for the four major included manufacturers. A comparison of curves for 5-year explantation risk was made with the log-rank (Mantel-Cox) test. (A) Explantation for any cause (p=0.11) and (B) explantation due to diminished pain relief (p=0.15).

To further examine the data on a more homogenous sample, a subgroup analysis was made of the 5-year explantation risk between the manufacturers, including 241 patients with PSPS2, which showed a higher risk for Medtronic that was not statistically significant (figure 3).

## Discussion

In this retrospective analysis, including 400 patients with stimulation devices implanted to treat chronic neuropathic pain, we present the overall explantation risk up to 10 years based on real-world data. The results show a cumulative risk of explantation for any reason of 17%, 23% and 38% at 3, 5 and 10 years, respectively. More importantly, explantation risk due to diminished pain relief at the same intervals was calculated to be 10%, 14% and 23%. Subgroup analysis of 5-year explantation risk for the four manufacturers revealed a possible higher risk of explantation for Medtronic SCS devices and DRG stimulation devices.

The explantation rates vary widely across different studies, ranging from 4% to 40%^1518^,^28^ and often differ based on the duration of the follow-up period. For a better understanding of the durability of SCS treatment over time, more studies with long-term follow-up are warranted.^29^ It is important to differentiate between explantation due to diminished pain relief and explantation for other reasons. We found that diminished pain relief was the most common reason for explantation, which occurred in over half of the patients. This observation is similar to findings in other studies.^19 24 26 30^

A factor complicating the comparison of explantation rates and risk with other studies is the discrepancy in how the data is presented. The included conditions treated with SCS may vary between studies and the duration of follow-up has not always been clearly defined. Moreover, in some studies, the number of explantations is simply divided by the number of implants without accounting for differences in follow-up duration, confusing rate with risk. With this in mind, the crude long-term explantation risk for any reason in our study is higher than described by Al-Kaisy^19^ and Slyer,^31^ but in the range of the findings by Van Buyten,^26^ Simopoulus,^23^ Teton^18^ and Hayek.^24^ Similarly, the risk of explantation due to diminished pain relief was higher than described by Al-Kaisy,^19^ but similar to Van Buyten^26^ and a recent study by Kirketeig.^15^ It is important to stress that explantation due to other reasons than diminished pain relief does not mean failure of the intention for the therapy. Nearly 20% of the explantations were due to the need for MRI (table 1), a number that will probably decline with increased use of full-body MRI conditional neuromodulation systems. The development of more robust leads also has the potential to minimize the risk of lead breakage or dysfunction of lead contacts with high impedances as a cause of explantation when an MRI is required.

To our knowledge, there are no studies investigating and comparing the explantation risks of devices from different SCS manufacturers. Previous studies have primarily explored how new SCS waveforms and stimulation modes compare with traditional tonic SCS in terms of efficacy to reduce pain and improve QoL and functional status. No comparison has been made in this respect or regarding explantation due to the loss of efficacy in the long term between new stimulation forms, such as various forms of burst stimulation, 10 kHz high-frequency stimulation, multimodal stimulation paradigms and DRG stimulation, supplied by different manufacturers. Moreover, data on explantation rates and risk is an efficient, objective outcome measure to demonstrate how SCS performs from a longer perspective. With the introduction of waveforms supplying paresthesia-free stimulation, which is preferred by many patients, we have gradually shifted to manufacturers that supply this type of stimulation in our practice. Therefore, we wanted to study the crude results regarding the 5-year explantation risk for neuromodulation devices from the four major manufacturers used in our department, with which we have extensive implanting and treating experience. In our data set, Kaplan-Meier and Cox regression analyses regarding explantation risk for any reason did not show a statistically different 5-year explantation risk between the manufacturers. However, in multivariable analysis of explantation risk due to diminished pain relief, a higher risk was seen for Medtronic SCS devices and for DRG stimulation, both in univariable and multivariable analyses.

Although not statistically significant in all analyses, it is interesting to speculate on the possible higher explantation risks seen in our material. For Medtronic, the probable explanation is that the majority of SCS devices were used at the beginning of the study period, rendering those systems less technically updated without the possibility of using new waveforms and paraesthesia-free stimulation and more often using devices that were not full-body MRI conditional. Moreover, our data show that the explantation risk for those manufacturers that for the majority of the study period supplied new SCS waveforms and stimulation paradigms seemed comparable. For Abbott, the increased explantation risk with DRG devices is in line with previous studies that indicate that DRG stimulation can be inflicted with rather high rates of lead breakage, dislocation and loss of function over time.^32^,^34^ Additionally, because different indications may influence the choice of an SCS system, we also performed a subgroup analysis of the 5-year explantation risk, including only the 241 patients with PSPS2. This analysis also showed a higher risk of explantation for Medtronic, which was not statistically significant.

Regarding other predictive factors for explantation, there are ambiguous reports in the literature. Conflicting results showing both higher and lower explantation risk when using rechargeable devices and/or high-frequency stimulation have been presented.^15 19 26 35^ Higher age^15^ and female gender^31^ have also been proposed as factors contributing to increased explantation risk. In our study, we did not find any other clinical variable to be a statistically significant predictor regarding explantation risk, including diabetes and immunosuppression treatment.

## Limitations

Several limitations regarding this study must be considered. Since it is a retrospective design, some data in medical records may be missing or difficult to find, which might lead to an inadequate presentation of the patient population and inaccurate results in the statistical analysis. We tried to minimize this risk by having two authors extract and cross-check all the available data. There are also several other variables than those included in this study that could be associated with explantation, which we have not accounted for, such as opioid treatment and psychosocial ill-being. In this study, we chose explantation as an objective outcome measure and have not addressed the frequency of, for example, revisions, which account for a large part of the complications seen in SCS treatment. The comparative analyses between the manufacturers that were close to a significant threshold call for caution regarding interpreting the results, since there is a risk for both type 1 error (a found difference that is not true) and type 2 error due to lack of power (falsely noting there is no difference because of too few included patients). It is important to note that during the study period, manufacturers have shifted technologies and waveforms and as such, comparing traditional (Medtronic) with modern SCS systems may be arguable. Our results, however, indicate an impact of technological improvement on better clinical outcomes and reduced explantation rates. Larger, prospective future studies investigating modern devices and leads with compiled data from independent neuromodulation registries are needed to enhance knowledge regarding explantation risks and causes. In addition, adherence to guidelines for appropriate referral and selection of patients with chronic pain for SCS based on multidisciplinary evaluation and recommendations, such as the recently developed SCS e-health tool (https://www.scstool.org/), has the potential to minimize long-term treatment failures.^1^

## Conclusions

Explantation of neuromodulation systems for pain treatment is not uncommon, and ineffective pain relief is the most frequent cause leading to explantation. Our data indicate that the explantation risk may vary based on the type of stimulation provided. However, the results were not fully statistically significant and more large, independent studies, not funded by the manufacturers providing SCS systems, are needed to confirm any differences in explantation risks over time and to define predictors associated with increased risk for explantation. Increased stringency in patient selection and follow-up strategies, as well as further improvement of software and hardware in neuromodulation devices, could possibly reduce the long-term explantation rates.

## Data Availability

Data are available upon reasonable request.
